# 2-Azido-*N*-(*o*-tol­yl)acetamide

**DOI:** 10.1107/S2414314626010503

**Published:** 2026-10-30

**Authors:** Abderrazzak El Moutaouakil Ala Allah, Musa A. Said, Vera Lederer, Biprajit Sarkar, Abdulsalam Alsubari, Joel T. Mague, Youssef Ramli

**Affiliations:** a Laboratory of Medicinal Chemistry Drug Sciences Research Center Faculty of Medicine and Pharmacy Mohammed V University in Rabat Morocco; b Department of Chemistry Faculty of Science Islamic University of Madinah, Madinah 42351 Saudi Arabia; c Institut fur Chemie und Biochemie Anorganische Chemie Freie Universität Berlin Fabeckstrasse 34-36 14195 Berlin Germany; d Laboratory of Medicinal Chemistry, Faculty of Clinical Pharmacy, 21 September University for Medical and Applied Sciences, Yemen; e Department of Chemistry, Tulane University, New Orleans, LA 70118, USA; Katholieke Universiteit Leuven, Belgium

**Keywords:** crystal structure, azide, hydrogen bond, acetamide, C—H⋯π(ring) inter­action, Hirshfeld surface

## Abstract

In the title mol­ecule, C_9_H_9_N_3_O, the inner C—N—C—C portion is almost planar and the phenyl ring and azide group are rotated to be well out of this plane. In the crystal, N—H⋯O and C—H⋯O hydrogen bonds plus C—H⋯π(ring) inter­actions form chains of mol­ecules extending along the *a*-axis direction, which pack with normal van der Waals inter­actions.

## Abstract 2

In the title mol­ecule, the inner C—N—C—C portion is almost planar and the phenyl ring and azide group are rotated to be well out of this plane. In the crystal, N—H⋯O and C—H⋯O hydrogen bonds plus C—H⋯π(ring) inter­actions form chains of mol­ecules extending along the *a*-axis direction.

## Graphical abstract

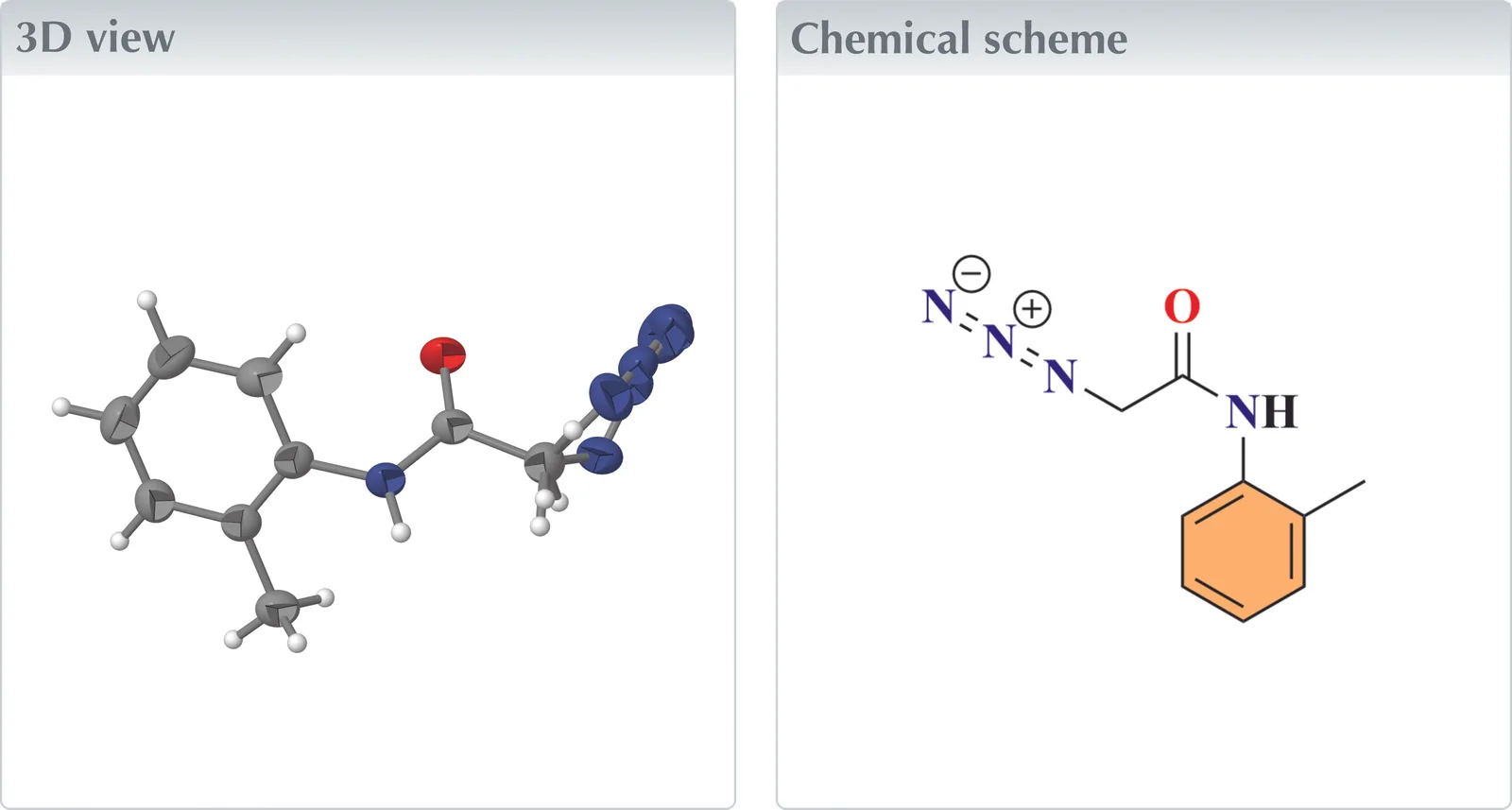

## Structure description

**Figure 1:**
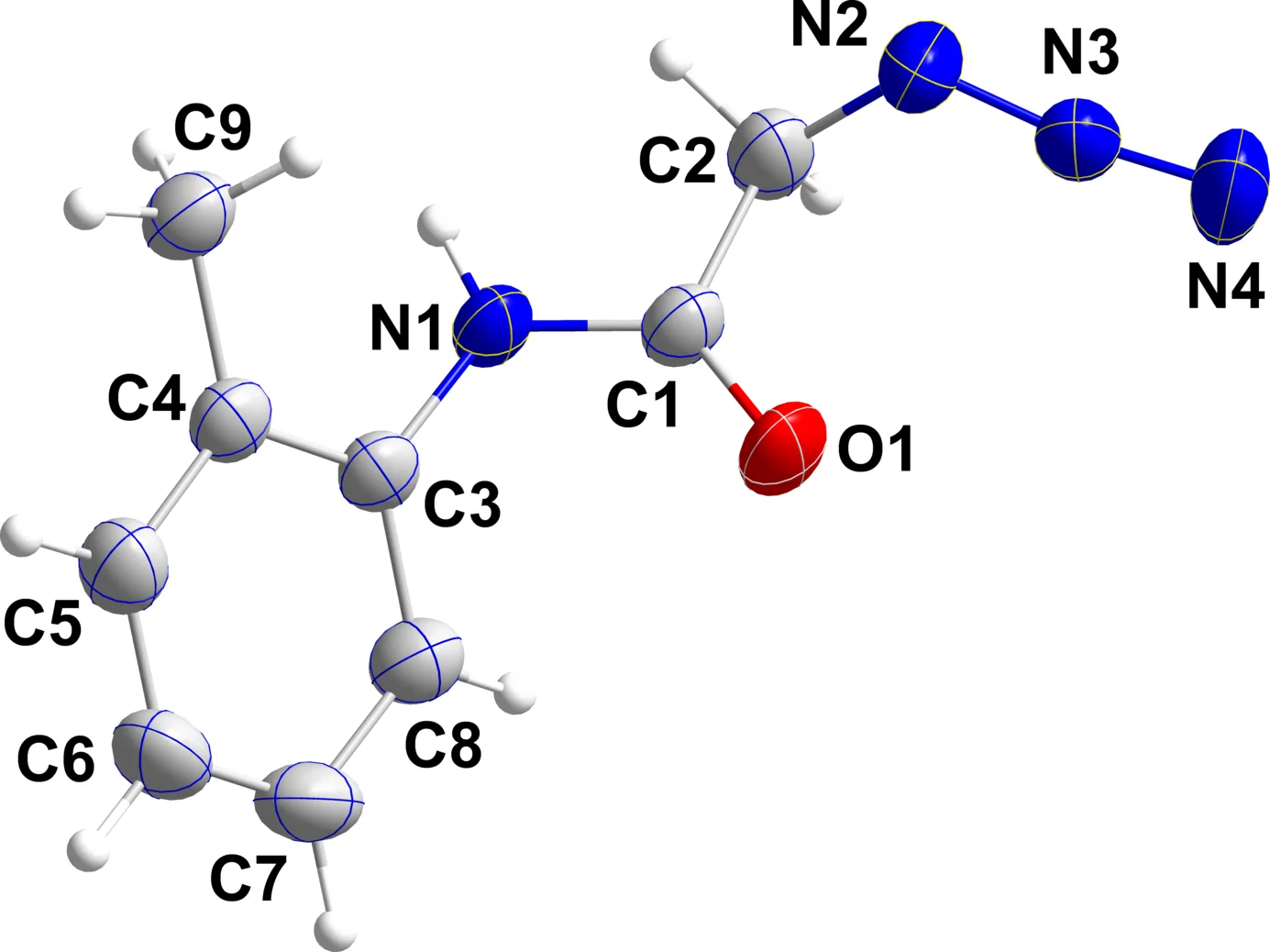
Perspective view of the title mol­ecule with labeling scheme and 50% probability ellipsoids. Only the major orientation of the azide group is shown.

A main target in medicinal chemistry is heo synthesis of compounds or libraries of compounds through a variety of chemical reactions during the drug discovery process (Patrick, 2023 ▸). Among the numerous synthetic methodologies available, the 1,3-dipolar cyclo­addition, also known as the Huisgen cyclo­addition, is widely employed for the construction of bioactive mol­ecules (Huisgen *et al.*, 1967 ▸). This reaction involves the cyclo­addition of an organic azide with a terminal alkyne, leading to the formation of 1,4- or 1,5-disubstituted 1,2,3-triazoles, depending on the catalyst and reaction conditions employed (Johansson *et al.*, 2016 ▸). Alkyl azides are regarded as key synthetic inter­mediates in medicinal chemistry because they provide efficient access to 1,2,3-triazole derivatives, a privileged heterocyclic scaffold that has been extensively exploited in the development of therapeutic agents for the treatment of a wide range of diseases (Zhou & Wang, 2012 ▸). With this objective in mind, and as a continuation of our ongoing research (*e.g.* Guerrab *et al.*, 2025 ▸), we report herein the synthesis and structural characterization of the title compound, C_9_H_9_N_3_O (**2**), prepared *via* nucleophilic substitution of the corresponding *N*-aryl chloro­acetamide precursor with sodium azide. The molecular structure of **2** is shown in Fig. 1 ▸.

The C3—N1—C1—C2 portion is planar within experimental error [torsion angle C3—N1—C1—C2 is −179.52 (14)°]. Both the azide and phenyl moieties are rotated out of this plane as indicated by the C1—N1—C3—C4 torsion angle of 131.37 (16)° and the dihedral angle between the amide group (O1/C1/N1) and benzene ring (C3–C8) of 48.4 (2)°. Bond lengths and inter­bond angles are as expected for the formulation given.

**Figure 2:**
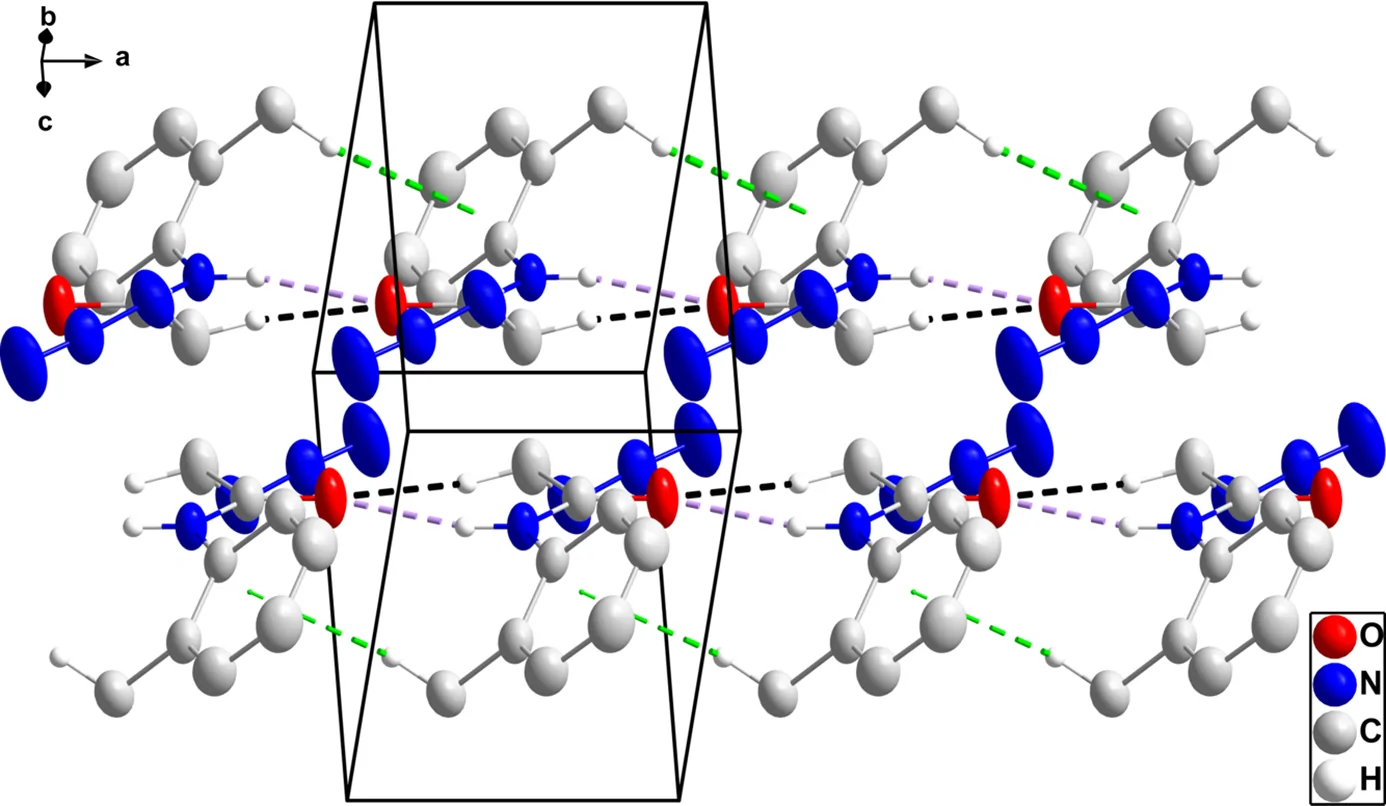
Packing projected onto (011) with N —H⋯O and C—H⋯O hydrogen bonds depicted, respectively, by violet and black dashed lines. The C—H⋯π(ring) inter­actions are depicted by green dashed lines and non-inter­acting hydrogen atoms are omitted for clarity.

In the crystal, N1—H1⋯O1^i^ and C2—H2AB⋯O1^i^ hydrogen bonds plus C9—H9*C*⋯*Cg*1^i^ inter­actions (*Cg*1 is the centroid of the C3–C8 ring; Table 1 ▸) form chains of mol­ecules extending along the *a*-axis direction (Fig. 2 ▸). The chains pack with normal van der Waals inter­actions.

**Figure 3:**
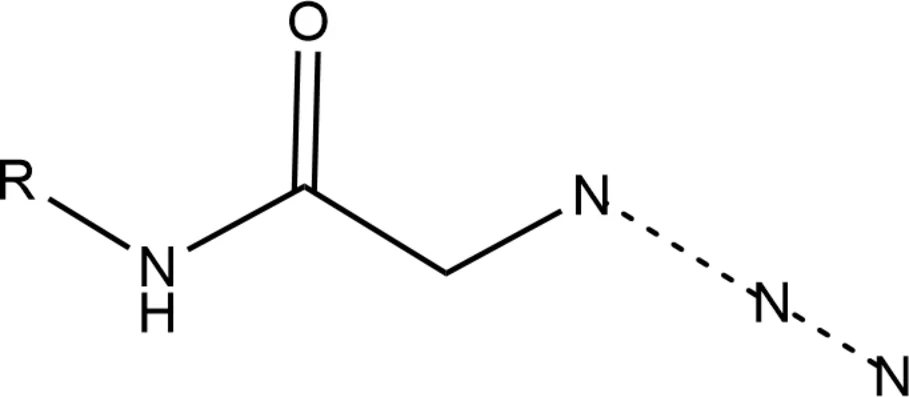
The search fragment used for the CSD database survey (*R* = C).

**Figure 4:**
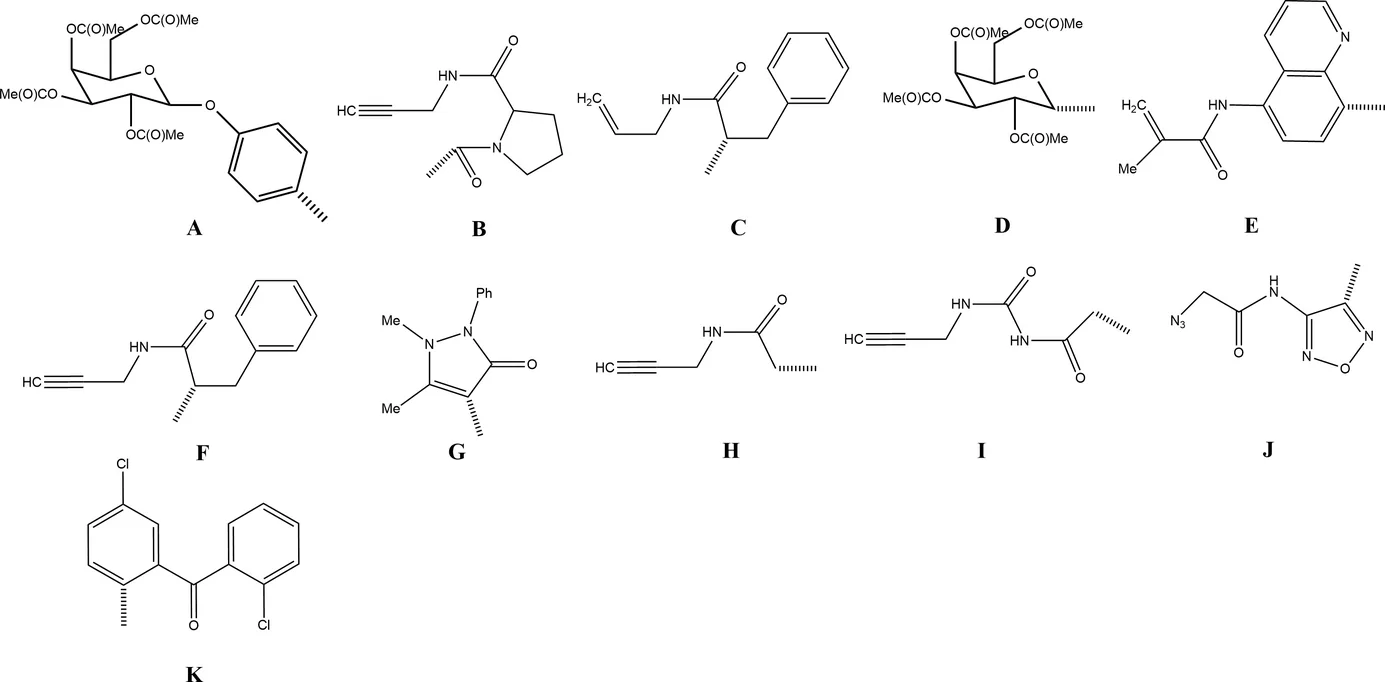
The substituents (*R*) referred to in Table 2 ▸. The wide dashed bond indicates the point of attachment.

A search of the Cambridge Structural Database (CSD, version 6.01, updated to June 2026; Groom *et al.*, 2016 ▸) with the fragment shown in Fig. 3 ▸ (*R* = C) yielded 85 hits. Many of these contained the N_3_ fragment as part of a triazole or a tetra­zole ring, which left 20 that are comparable to the title mol­ecule. These are listed in Table 2 ▸ with the more complex *R* groups pictured in Fig. 4 ▸. Inspection of the torsion angles corresponding to the N1—C1—C2—N2 and C1—N1—C3—C4 torsion angles in the title mol­ecule (Table 2 ▸) shows that in the similar mol­ecules, the conformations vary widely. Since the *R* groups vary greatly in size and shape, this is not surprising and likely can be attributed to packing considerations.

**Figure 5:**
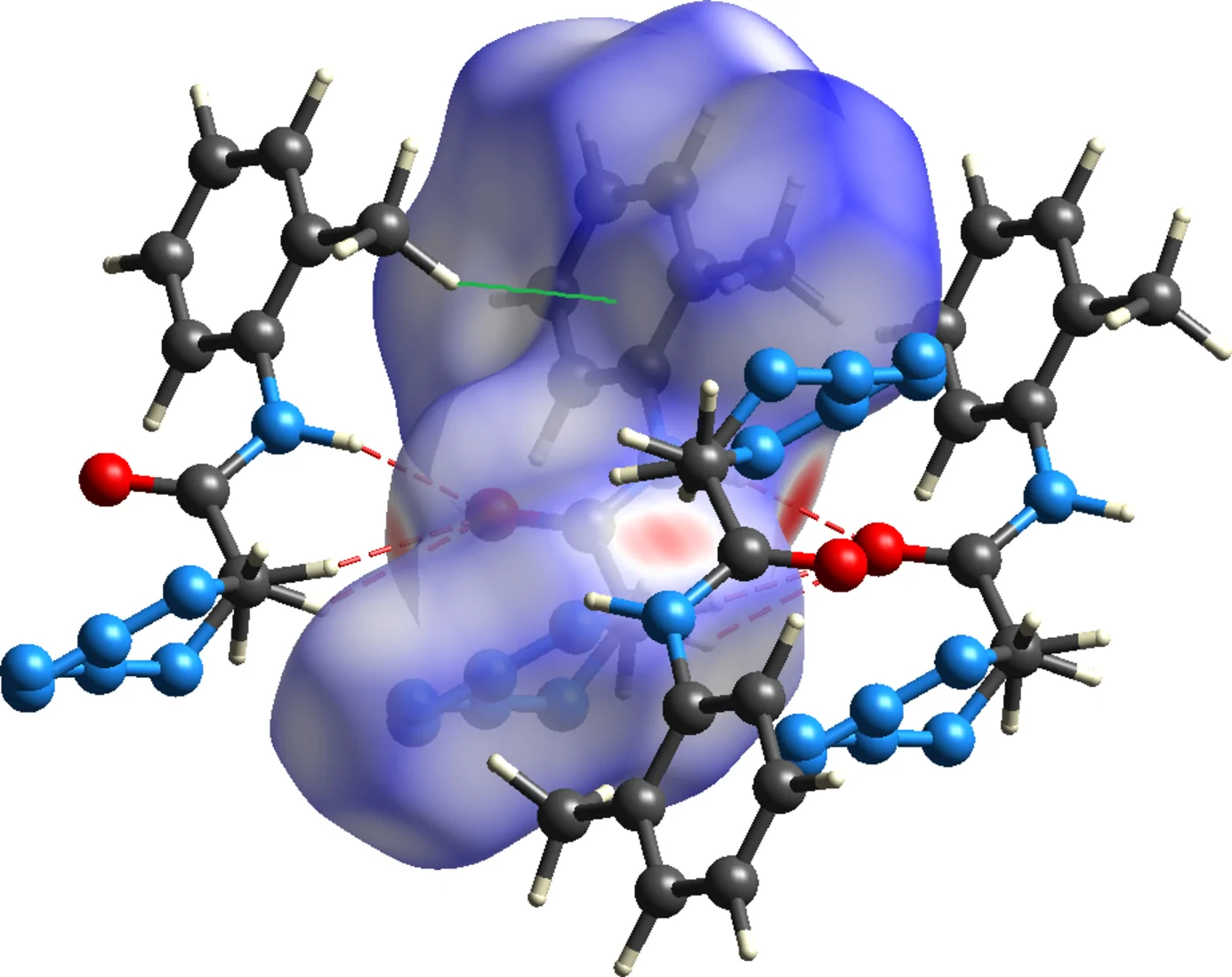
The *d*_norm_ Hirshfeld surface of the title mol­ecule together with neighboring mol­ecules and indicating the hydrogen bonds (red dashed lines) and C—H⋯π(ring) inter­actions (green line).

**Figure 6:**
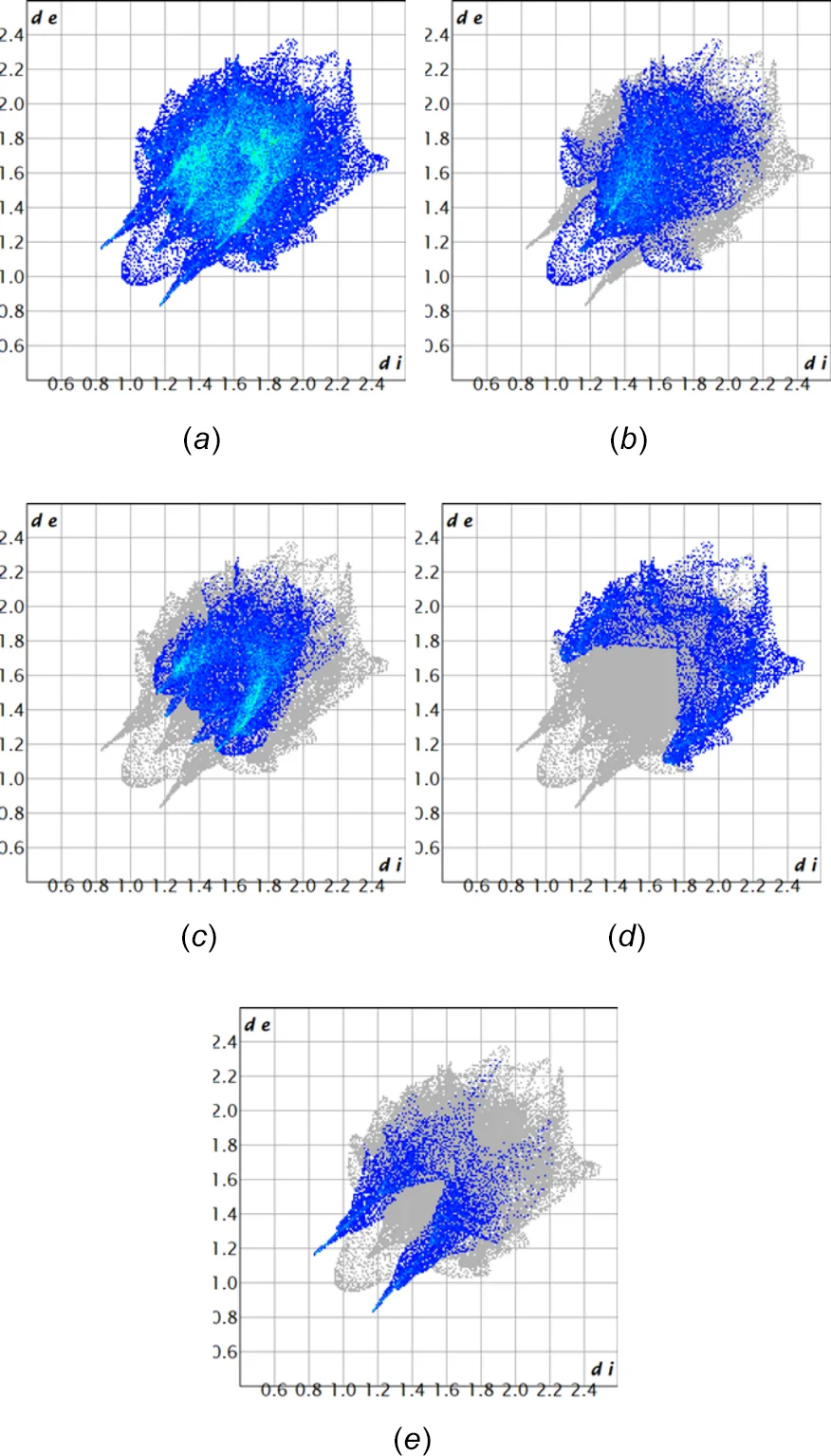
Two-dimensional fingerprint plots showing all inter­molecular inter­actions (*a*), the H⋯H inter­actions (*b*), the N⋯H/H⋯N inter­actions (*c*), the C⋯H/H⋯C inter­actions (*d*) and the O⋯H/H⋯O inter­actions (*e*).

The *d*_norm_ Hirshfeld surface of the title mol­ecule was calculated with *CrystalExplorer* (Spackman *et al.*, 2021 ▸) and is shown in Fig. 5 ▸. Descriptions of the plots obtained by this method and their inter­pretations have been published (Tan *et al.*, 2019 ▸). In Fig. 5 ▸, the intense red spots indicate the locations of the N—H⋯O hydrogen bonds to the neighboring mol­ecules while the C—H⋯O hydrogen bonds are indicated by lighter red spots and the C—H⋯π(ring) inter­action by the green line. The two-dimensional fingerprint plots are shown in Fig. 6 ▸ with all inter­actions in Fig. 6 ▸*a*, and the H⋯H inter­actions in Fig. 6 ▸*b*. The latter contribute 32.6% of the total inter­molecular contacts which is often the case for organic mol­ecules whose periphery consists largely of hydrogen atoms. It might be surprising that the N⋯H/H⋯N contacts (Fig. 6 ▸*c*) comprise 33.3% of the total since there are no directed hydrogen bonds to nitro­gen. However, with the considerable nitro­gen content due to the azide group, there are six N⋯H contacts at or close to the sum of the respective van der Waals radii. The other significant contributions come from the C⋯H/H⋯C (Fig. 6 ▸*d*, 17.3%) and the O⋯H/H⋯O (Fig. 6 ▸*e*, 10.5%) contacts. The former are due primarily to the C—H⋯π(ring) inter­actions and appear as a pair of rather blunt peaks. The latter appear as a pair of sharp peaks attributed to the N—H⋯O hydrogen bonds with broader shoulders which correspond to the C—H⋯O hydrogen bonds.

## Synthesis and crystallization

The precursor 2-chloro-*N*-(*o*-tol­yl)acetamide (**1**) was prepared according to the procedure described in our previous work (*e.g.* El Moutaouakil Ala Allah *et al.*, 2024 ▸). A mixture of 2.73 mmol of compound **1** and 3.52 mmol of sodium azide was dissolved in an ethanol/water mixture and refluxed at 80 °C for 24 h. Upon completion of the reaction, as monitored by thin-layer chromatography (TLC), the precipitated 2-azido-*N*-(*o*-tol­yl)acetamide (**2**) was collected by filtration and washed with cold water. The resulting solid was subsequently recrystallized from ethanol. Single crystals suitable for X-ray diffraction analysis were obtained by slow evaporation from ethanol solution.

Yield 82%, white solid, m.p. = 455–457 K. FT–IR (ATR, cm^−1^): 3218 (NH), 2110 (N_3_), 1640 (C=O); ^1^H NMR (500 MHz, DMSO-*d_6_*): δ p.p.m. 4.18 (*s*, 2H, CH_2_), 4.32 (*s*, 3H, CH_3_), 6.72–7.30 (*m*, 4H, Ar—H), 10.82 (*s*, 1H, NH); ^13^C NMR: 44.25 (CH_2_), 48.23 (CH_3_), 118.62, 121.25, 134.62, 148.59 (C—Ar); 169.23 (C=O).

## Refinement

Crystal data, data collection and structure refinement details are summarized in Table 3 ▸. The azide group is disordered over two sites related by a 22.4 (3)° rotation about the C1—C2 bond in a 0.740 (4)/0.260 (4) ratio. The two components were refined with restraints that their geometries be comparable.

## Supplementary Material

Crystal structure: contains datablock(s) I. DOI: 10.1107/S2414314626010503/vm4085sup1.cif

Structure factors: contains datablock(s) I. DOI: 10.1107/S2414314626010503/vm4085Isup2.hkl

Supporting information file. DOI: 10.1107/S2414314626010503/vm4085Isup3.cml

CCDC reference: 2594679

Additional supporting information:  crystallographic information; 3D view; checkCIF report

## Figures and Tables

**Table 1:** Hydrogen-bond geometry (Å, °) *Cg*1 is the centroid of the C3–C8 benzene ring.

*D*—H⋯*A*	*D*—H	H⋯*A*	*D*⋯*A*	*D*—H⋯*A*
N1—H1⋯O1^i^	0.87 (2)	2.14 (2)	2.9918 (15)	166 (2)
C2—H2*AB*⋯O1^i^	0.98	2.37	3.2576 (19)	150
C9—H9*C*⋯*Cg*1^i^	0.97	2.81	3.5817 (18)	137

Symmetry code: (i) 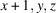.

**Table 2:** Results of CSD database survey

Compound	*R* ^*a*^	Torsion 1^*b*^ (°)	Torsion 2^*c*^ (°)	Reference
Title mol­ecule	*o*-tol­yl	132.74 (16)	131.37 (16)	This work
ASEDIO	phen­yl	169.90 (18), 160.9 (2)	151,3(3), 161.5 (2)	Guerrab *et al.* (2021 ▸)
BEBPIJ	A	−17.4 (13)	−164.4 (10)	Cecioni *et al.* (2012 ▸)
BEKRES	*p*-tol­yl	−177.75 (18), −176.64 (18), −179.23 (18)	−156.8 (2), −160.2 (2), −158.8 (2)	Missioui *et al.* (2022*a* ▸)
BEKRIW	4-FC_6_H_4_	167.98 (18), −156.64 (18)	−158.4 (2), −158.8 (2)	Missioui *et al.* (2022*b* ▸)
CUVVEY	2,6-Me_2_C_6_H_3_	−137.3 (5), 136.6((4)	−116.5 (6), 114.9 (5)	El Moutaouakil Ala Allah *et al.* (2025 ▸)
DAPYOU	4-HC_2_C_6_H_4_	−160.0 (4), −175.4 (3)	−176.2 (4), 168.4 (3)	Madhusudhanan *et al.* (2021 ▸)
GUYCUC	B	103.7 (9)	−78.2 (6)	Rai *et al.* (2025 ▸)
HIQKIF	C	−145.6 (4)	−126.0 (3)	Khazeber & Sureshan (2022 ▸)
KEPKOG	D	6.5 (2)	126.8 (4)	Petsalakis *et al.* (2006 ▸)
LETTIR	E	5.7 (3)	1.6 (4)	Guirado-Moreno *et al.* (2023 ▸)
PADGEK	F	166.5 (7)	118.6 (6)	Hema & Sureshan (2020 ▸)
QAGNOF	4-O_2_NC_6_H_4_	142.68 (13)	15.99 (19)	Missioui *et al.* (2020 ▸)
QIMYOE	G	123.53 (9)	68.76 (12)	Kumar *et al.* (2023 ▸)
TARHIH	4-MeOC_6_H_4_	125.25 (14)	156.34 (15)	Missioui *et al.* (2022*c* ▸)
TODFIZ	H	−161.8 (8)	−173.9 (8)	Athiyarath & Sureshan (2019 ▸)
TODVAM	I	161.9 (4)	178.0 (4)	Athiyarath & Sureshan (2019 ▸)
TUZPEJ	J	10.0 (3), 176.0 (2)	146.9 (3), 37.3 (4)	Li *et al.* (2003 ▸)
VIFVOX	K	−170.3 (3), −3.8 (4)	161.9 (2), 174.7 (2)	Sandra *et al.* (2012 ▸)
WOQBOX	PhCH_2_CH_2_	177.98 (13)	84.67 (17)	Cedillo-Cruz *et al.* (2024 ▸)

Notes: (*a*) Letters refer to the drawings in Fig. 4 ▸; (*b*) corresponds to the N1—C1—C2—N2 torsion in the title mol­ecule; (*c*) corresponds to the C1—N1—C3—C4 torsion in the title mol­ecule.

**Table 3:** Experimental details

Crystal data	
Chemical formula	C_9_H_10_N_4_O
*M*_r_	190.21
Crystal system, space group	Triclinic, *P* 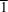
Temperature (K)	250
*a*, *b*, *c* (Å)	4.8642 (6), 9.9359 (13), 10.4042 (9)
α, β, γ (°)	70.153 (10), 87.279 (8), 84.833 (10)
*V* (Å^3^)	470.97 (10)
*Z*	2
Radiation type	Cu *K*α
μ (mm^−1^)	0.77
Crystal size (mm)	0.46 × 0.16 × 0.13

Data collection	
Diffractometer	SuperNova, Dual, Cu at home/near, Atlas
Absorption correction	Gaussian (*CrysAlis PRO*; Rigaku OD, 2026 ▸)
*T*_min_, *T*_max_	0.474, 1.000
No. of measured, independent and observed [*I* > 2σ(*I*)] reflections	2964, 1787, 1446
*R*_int_	0.018
(sin θ/λ)_max_ (Å^−1^)	0.619

Refinement	
*R*[*F*^2^ > 2σ(*F*^2^)], *wR*(*F*^2^), *S*	0.039, 0.116, 1.05
No. of reflections	1787
No. of parameters	159
No. of restraints	48
H-atom treatment	H atoms treated by a mixture of independent and constrained refinement
Δρ_max_, Δρ_min_ (e Å^−3^)	0.22, −0.16

Computer programs: *CrysAlis PRO* (Rigaku OD, 2026 ▸), *SHELXT2018/2* (Sheldrick, 2015*a* ▸), *SHELXL2018/3* (Sheldrick, 2015*b* ▸) and *OLEX2* (Dolomanov *et al.*, 2009 ▸).

## full crystallographic data

### 2-Azido-N-(2-methylphenyl)acetamide Crystal data

**Table:**

C_9_H_10_N_4_O	*Z* = 2
*M**_r_* = 190.21	*F*(000) = 200
Triclinic, *P*1	*D*_x_ = 1.341 Mg m^−^^3^
*a* = 4.8642 (6) Å	Cu *K*α radiation, λ = 1.54184 Å
*b* = 9.9359 (13) Å	Cell parameters from 1534 reflections
*c* = 10.4042 (9) Å	θ = 4.5–72.6°
α = 70.153 (10)°	µ = 0.77 mm^−^^1^
β = 87.279 (8)°	*T* = 250 K
γ = 84.833 (10)°	Plank, colourless
*V* = 470.97 (10) Å^3^	0.46 × 0.16 × 0.13 mm

### 2-Azido-N-(2-methylphenyl)acetamide Data collection

**Table:**

SuperNova, Dual, Cu at home/near, Atlas diffractometer	1787 independent reflections
Radiation source: micro-focus sealed X-ray tube, SuperNova (Cu) X-ray Source	1446 reflections with *I* > 2σ(*I*)
Mirror monochromator	*R*_int_ = 0.018
Detector resolution: 10.5082 pixels mm^-1^	θ_max_ = 72.8°, θ_min_ = 4.5°
ω scans	*h* = −5→5
Absorption correction: gaussian (CrysAlisPro; Rigaku OD, 2026)	*k* = −11→12
*T*_min_ = 0.474, *T*_max_ = 1.000	*l* = −7→12
2964 measured reflections	

### 2-Azido-N-(2-methylphenyl)acetamide Refinement

**Table:**

Refinement on *F*^2^	Primary atom site location: dual
Least-squares matrix: full	Hydrogen site location: mixed
*R*[*F*^2^ > 2σ(*F*^2^)] = 0.039	H atoms treated by a mixture of independent and constrained refinement
*wR*(*F*^2^) = 0.116	*w* = 1/[σ^2^(*F*_o_^2^) + (0.0606*P*)^2^ + 0.0544*P*] where *P* = (*F*_o_^2^ + 2*F*_c_^2^)/3
*S* = 1.05	(Δ/σ)_max_ < 0.001
1787 reflections	Δρ_max_ = 0.22 e Å^−^^3^
159 parameters	Δρ_min_ = −0.16 e Å^−^^3^
48 restraints	

### 2-Azido-N-(2-methylphenyl)acetamide Special details

**Table:**

Geometry. All esds (except the esd in the dihedral angle between two l.s. planes) are estimated using the full covariance matrix. The cell esds are taken into account individually in the estimation of esds in distances, angles and torsion angles; correlations between esds in cell parameters are only used when they are defined by crystal symmetry. An approximate (isotropic) treatment of cell esds is used for estimating esds involving l.s. planes.
Refinement. The crystals contain a disordered azide moiety. The azide consist of N atoms N2-4 (Part 1) and N2A-4A (Part 2) The distances of the disordered groups were restrained to be similar (SADI) and the thermal parameters were also restrained (SIMU, RIGU. The occupancies were allowed to freely refine to sum unity, with a ratio of Part1:part2 of 0.74 (1):0.26 (1).All hydrogen atoms except H1 were observed in the electron density map before placing and refining using a riding model. Hydrogen atom H1 was refined and the N-H distance restrained to a target value of 0.88 %A (DFIX). The isotropic dispalcement parameters of hydrogen atom H1 was fixed at a value of 1.2 Ueq of the parent nitrogen atom N1.

### 2-Azido-N-(2-methylphenyl)acetamide Fractional atomic coordinates and isotropic or equivalent isotropic displacement parameters (Å^2^)

**Table:**

	*x*	*y*	*z*	*U*_iso_*/*U*_eq_	Occ. (<1)
O1	0.04695 (19)	0.74648 (14)	0.48779 (12)	0.0551 (3)	
N1	0.4802 (2)	0.72303 (14)	0.40158 (12)	0.0400 (3)	
N3	0.0729 (8)	0.9021 (4)	0.6931 (5)	0.0494 (9)	0.740 (4)
C3	0.4099 (3)	0.68309 (16)	0.28864 (14)	0.0374 (3)	
C1	0.2968 (3)	0.75172 (17)	0.49098 (14)	0.0400 (3)	
C4	0.5213 (3)	0.75306 (16)	0.15991 (15)	0.0395 (3)	
C8	0.2337 (3)	0.57458 (17)	0.30866 (16)	0.0458 (4)	
H8	0.163675	0.526827	0.396628	0.055*	
C5	0.4474 (3)	0.71126 (19)	0.05240 (17)	0.0493 (4)	
H5	0.520754	0.756276	−0.035484	0.059*	
C9	0.7116 (3)	0.87048 (18)	0.13746 (16)	0.0475 (4)	
H9A	0.619476	0.946175	0.166719	0.071*	
H9B	0.760659	0.909159	0.041157	0.071*	
H9C	0.877472	0.831851	0.190031	0.071*	
N4	−0.1164 (15)	0.9030 (10)	0.7588 (10)	0.081 (2)	0.740 (4)
C7	0.1620 (3)	0.53715 (19)	0.19932 (19)	0.0548 (4)	
H7	0.040316	0.465146	0.212335	0.066*	
C2	0.4226 (3)	0.7929 (2)	0.60148 (17)	0.0516 (4)	
H2AA	0.417820	0.712307	0.687708	0.062*	0.740 (4)
H2AB	0.616616	0.810274	0.577377	0.062*	0.740 (4)
H2BC	0.603671	0.739849	0.623958	0.062*	0.260 (4)
H2BD	0.452032	0.895441	0.565423	0.062*	0.260 (4)
C6	0.2689 (3)	0.6053 (2)	0.07137 (18)	0.0557 (4)	
H6	0.220633	0.579867	−0.003277	0.067*	
N2	0.2826 (4)	0.9194 (2)	0.6216 (2)	0.0573 (7)	0.740 (4)
N2A	0.2616 (11)	0.7659 (7)	0.7216 (5)	0.0549 (17)	0.260 (4)
N3A	0.065 (2)	0.8486 (12)	0.7275 (15)	0.053 (3)	0.260 (4)
N4A	−0.121 (5)	0.921 (3)	0.740 (3)	0.087 (6)	0.260 (4)
H1	0.651 (4)	0.737 (3)	0.412 (2)	0.104*	

### 2-Azido-N-(2-methylphenyl)acetamide Atomic displacement parameters (Å^2^)

**Table:**

	*U*^11^	*U*^22^	*U*^33^	*U*^12^	*U*^13^	*U*^23^
O1	0.0242 (5)	0.0869 (9)	0.0647 (7)	−0.0092 (5)	0.0024 (5)	−0.0385 (7)
N1	0.0240 (6)	0.0584 (8)	0.0420 (7)	−0.0063 (5)	−0.0007 (5)	−0.0219 (6)
N3	0.0376 (14)	0.067 (2)	0.057 (2)	−0.0111 (16)	0.0033 (12)	−0.0374 (19)
C3	0.0257 (6)	0.0461 (8)	0.0435 (8)	−0.0003 (5)	−0.0042 (5)	−0.0194 (6)
C1	0.0279 (7)	0.0510 (8)	0.0430 (8)	−0.0059 (6)	0.0001 (5)	−0.0177 (6)
C4	0.0283 (7)	0.0478 (8)	0.0449 (8)	−0.0012 (6)	−0.0014 (5)	−0.0190 (6)
C8	0.0394 (8)	0.0478 (9)	0.0509 (9)	−0.0082 (6)	−0.0004 (6)	−0.0162 (7)
C5	0.0450 (9)	0.0629 (10)	0.0441 (8)	−0.0051 (7)	−0.0003 (6)	−0.0231 (7)
C9	0.0380 (8)	0.0573 (10)	0.0479 (8)	−0.0112 (7)	0.0014 (6)	−0.0168 (7)
N4	0.052 (3)	0.131 (5)	0.083 (3)	−0.024 (2)	0.029 (2)	−0.067 (3)
C7	0.0497 (9)	0.0538 (10)	0.0700 (11)	−0.0138 (7)	−0.0051 (8)	−0.0296 (8)
C2	0.0357 (8)	0.0760 (12)	0.0540 (9)	−0.0089 (7)	0.0023 (6)	−0.0356 (9)
C6	0.0528 (10)	0.0685 (11)	0.0591 (10)	−0.0068 (8)	−0.0072 (8)	−0.0375 (9)
N2	0.0442 (11)	0.0663 (14)	0.0723 (14)	−0.0169 (9)	0.0196 (9)	−0.0370 (11)
N2A	0.051 (3)	0.073 (4)	0.042 (3)	−0.003 (3)	0.002 (2)	−0.021 (3)
N3A	0.045 (4)	0.071 (6)	0.058 (7)	−0.021 (4)	0.006 (4)	−0.037 (5)
N4A	0.070 (10)	0.083 (8)	0.130 (16)	0.008 (6)	−0.010 (8)	−0.068 (9)

### 2-Azido-N-(2-methylphenyl)acetamide Geometric parameters (Å, º)

**Table:**

O1—C1	1.2234 (16)	C9—H9A	0.9700
N1—C3	1.4263 (17)	C9—H9B	0.9700
N1—C1	1.3408 (18)	C9—H9C	0.9700
N1—H1	0.873 (16)	C7—H7	0.9400
N3—N4	1.122 (6)	C7—C6	1.374 (3)
N3—N2	1.223 (4)	C2—H2AA	0.9800
C3—C4	1.392 (2)	C2—H2AB	0.9800
C3—C8	1.392 (2)	C2—H2BC	0.9800
C1—C2	1.515 (2)	C2—H2BD	0.9800
C4—C5	1.391 (2)	C2—N2	1.449 (3)
C4—C9	1.504 (2)	C2—N2A	1.402 (5)
C8—H8	0.9400	C6—H6	0.9400
C8—C7	1.379 (2)	N2A—N3A	1.217 (11)
C5—H5	0.9400	N3A—N4A	1.136 (14)
C5—C6	1.381 (2)		
			
C3—N1—H1	120.0 (17)	H9A—C9—H9C	109.5
C1—N1—C3	124.46 (11)	H9B—C9—H9C	109.5
C1—N1—H1	115.4 (17)	C8—C7—H7	120.1
N4—N3—N2	171.4 (6)	C6—C7—C8	119.85 (15)
C4—C3—N1	119.13 (12)	C6—C7—H7	120.1
C8—C3—N1	119.80 (13)	C1—C2—H2AA	108.9
C8—C3—C4	121.07 (13)	C1—C2—H2AB	108.9
O1—C1—N1	124.94 (13)	C1—C2—H2BC	108.7
O1—C1—C2	120.59 (13)	C1—C2—H2BD	108.7
N1—C1—C2	114.47 (11)	H2AA—C2—H2AB	107.7
C3—C4—C9	121.38 (13)	H2BC—C2—H2BD	107.6
C5—C4—C3	117.40 (14)	N2—C2—C1	113.38 (15)
C5—C4—C9	121.22 (14)	N2—C2—H2AA	108.9
C3—C8—H8	120.0	N2—C2—H2AB	108.9
C7—C8—C3	119.95 (15)	N2A—C2—C1	114.1 (2)
C7—C8—H8	120.0	N2A—C2—H2BC	108.7
C4—C5—H5	119.1	N2A—C2—H2BD	108.7
C6—C5—C4	121.72 (15)	C5—C6—H6	120.0
C6—C5—H5	119.1	C7—C6—C5	119.99 (15)
C4—C9—H9A	109.5	C7—C6—H6	120.0
C4—C9—H9B	109.5	N3—N2—C2	116.9 (2)
C4—C9—H9C	109.5	N3A—N2A—C2	120.0 (7)
H9A—C9—H9B	109.5	N4A—N3A—N2A	176 (2)
			
O1—C1—C2—N2	−47.3 (2)	C1—N1—C3—C4	131.37 (16)
O1—C1—C2—N2A	24.8 (4)	C1—N1—C3—C8	−49.2 (2)
N1—C3—C4—C5	−179.88 (13)	C1—C2—N2—N3	81.8 (4)
N1—C3—C4—C9	−0.6 (2)	C1—C2—N2A—N3A	−79.6 (10)
N1—C3—C8—C7	179.04 (14)	C4—C3—C8—C7	−1.5 (2)
N1—C1—C2—N2	132.74 (16)	C4—C5—C6—C7	−0.7 (3)
N1—C1—C2—N2A	−155.1 (3)	C8—C3—C4—C5	0.6 (2)
C3—N1—C1—O1	0.5 (3)	C8—C3—C4—C9	179.96 (14)
C3—N1—C1—C2	−179.52 (14)	C8—C7—C6—C5	−0.1 (3)
C3—C4—C5—C6	0.5 (2)	C9—C4—C5—C6	−178.84 (15)
C3—C8—C7—C6	1.2 (3)		

### 2-Azido-N-(2-methylphenyl)acetamide Hydrogen-bond geometry (Å, º)

*Cg*1 is the centroid of the C3–C8 benzene ring.

**Table:**

*D*—H···*A*	*D*—H	H···*A*	*D*···*A*	*D*—H···*A*
N1—H1···O1^i^	0.87 (2)	2.14 (2)	2.9918 (15)	166 (2)
C2—H2*AB*···O1^i^	0.98	2.37	3.2576 (19)	150
C9—H9*C*···Cg1^i^	0.97	2.81	3.5817 (18)	137

Symmetry code: (i) *x*+1, *y*, *z*.

## References

[bb1] Athiyarath, V. & Sureshan, K. M. (2019). *Angew. Chem. Int. Ed.* **58**, 612–617. 10.1002/anie.201812094 30461147

[bb2] Cecioni, S., Praly, J.-P., Matthews, S. E., Wimmerová, M., Imberty, A. & Vidal, S. (2012). *Chem. Eur. J.* **18**, 6250–6263. 10.1002/chem.201200010 22488581

[bb3] Cedillo–Cruz, A., Villalobos–López, D. C., Zavala–Segovia, N., Aguilar, M. I. & Cuevas–Yañez, E. (2024). *J. Mol. Struct.* **1309**, 138178.

[bb4] Dolomanov, O. V., Bourhis, L. J., Gildea, R. J., Howard, J. A. K. & Puschmann, H. (2009). *J. Appl. Cryst.* **42**, 339–341.

[bb5] El Moutaouakil Ala Allah, A., Kariuki, B. M., Al-Sulami, A. I., Basha, M. T., Allehyani, B. H., Alsubari, A., Mague, J. T. & Ramli, Y. (2025). *Acta Cryst.* E**81**, 627–631. 10.1107/S2056989025004530 PMC12230608 40630657

[bb6] El Moutaouakil Ala Allah, A., Kariuki, B. M., Ameziane El Hassani, I., Alsubari, A., Guerrab, W., Said, M. & Ramli, Y. (2024). *IUCrData* **9**, x241015. 10.1107/S2414314624010150 PMC11660168 39712662

[bb7] Groom, C. R., Bruno, I. J., Lightfoot, M. P. & Ward, S. C. (2016). *Acta Cryst.* B**72**, 171–179. 10.1107/S2052520616003954 PMC4822653 27048719

[bb8] Guerrab, W., Missioui, M., Zaoui, Y., Mague, J. T. & Ramli, Y. (2021). *Z. Kristallogr. New Cryst. Struct.* **236**, 133–134.

[bb9] Guerrab, W., Mortada, S., El Moutaouakil Ala Allah, A., Demirtaş, G., Mague, J. T., Alzahrani, A. Y. A., AL Mughram, M. H., Faouzi, M. E. A. & Ramli, Y. (2025). *J. Mol. Struct.* **1333**, 141802.

[bb10] Guirado-Moreno, J. C., González-Ceballos, L., Carreira-Barral, I., Ibeas, S., Fernández-Muiño, M. A., Teresa Sancho, M., García, J. M. & Vallejos, S. (2023). *Spectrochim. Acta A Mol. Biomol. Spectrosc.* **284**, 121820. 10.1016/j.saa.2022.121820 36116204

[bb11] Hema, K. & Sureshan, K. M. (2020). *Angew. Chem. Int. Ed.* **59**, 8854–8859. 10.1002/anie.201914975 32149438

[bb12] Huisgen, R., Szeimies, G. & Möbius, L. (1967). *Chem. Ber.* **100**, 2494–2507.

[bb13] Johansson, J. R., Beke-Somfai, T., Said Stålsmeden, A. & Kann, N. (2016). *Chem. Rev.* **116**, 14726–14768. 10.1021/acs.chemrev.6b00466 27960271

[bb14] Khazeber, R. & Sureshan, K. M. (2022). *Proc. Natl Acad. Sci. USA* **119**, e2205320119. 10.1073/pnas.2205320119 PMC9303982 35858342

[bb15] Kumar, S., Lal, B., Tittal, R. K., Singh, G., Singh, J., Sharma, R. & Sabane, J. K. (2023). *Sens. Diagn.* **2**, 1267–1276.

[bb16] Li, Z.-X., Tang, S.-Q., Liu, J.-T. & Sun, J. (2003). *Jiegou Huaxue(Chin.)(Chin. J. Struct. Chem.)* **2**, 25.

[bb17] Madhusudhanan, M. C., Balan, H., Werz, D. B. & Sureshan, K. M. (2021). *Angew. Chem. Int. Ed.* **60**, 22797–22803. 10.1002/anie.202106614 34399025

[bb18] Missioui, M., Guerrab, W., Alsubari, A., Mague, J. T. & Ramli, Y. (2022*a*). *IUCrData* **7**, x220621. 10.1107/S2414314622006216 PMC9635410 36341048

[bb19] Missioui, M., Guerrab, W., Alsubari, A., Mague, J. T. & Ramli, Y. (2022*b*). *Acta Cryst.* E**78**, 855–859. 10.1107/S2056989022006764 PMC9361366 35974836

[bb20] Missioui, M., Guerrab, W., Mague, J. T. & Ramli, Y. (2020). *Z. Kristallogr. New Cryst. Struct.* **235**, 1429–1430.

[bb21] Missioui, M., Lgaz, H., Guerrab, W., Lee, H.-S., Warad, I., Mague, J. T., Ali, I. H., <Essassi, El, M. & Ramli, Y. (2022*c*). *J. Mol. Struct.* **1253**, 132132.

[bb22] Patrick, G. L. (2023). *An introduction to medicinal chemistry.* Oxford University Press.

[bb23] Petsalakis, E. I., Chrysina, E. D., Tiraidis, C., Hadjiloi, T., Leonidas, D. D., Oikonomakos, N. G., Aich, U., Varghese, B. & Loganathan, D. (2006). *Bioorg. Med. Chem.* **14**, 5316–5324. 10.1016/j.bmc.2006.03.044 16616506

[bb24] Rai, R., Xavier, D., Pathak, S., Fernandez, F. B., Komath, M. & Sureshan, K. M. (2025). *Angew. Chem. Int. Ed.* **64**, e202422593. 10.1002/anie.202422593 39791355

[bb25] Rigaku OD (2026). *CrysAlis PRO*. Rigaku Oxford Diffraction, Yarnton, England.

[bb26] Sandra, C.-M., Eduardo, C. C., Simon, H.-O., Teresa, R. A., Antonio, N. C., Lijanova, I. V. & Marcos, M.-G. (2012). *Anticancer Agents Med. Chem.* **12**, 611–618. 10.2174/187152012800617713 22263787

[bb27] Sheldrick, G. M. (2015*a*). *Acta Cryst.* A**71**, 3–8.

[bb28] Sheldrick, G. M. (2015*b*). *Acta Cryst.* C**71**, 3–8.

[bb29] Spackman, P. R., Turner, M. J., McKinnon, J. J., Wolff, S. K., Grimwood, D. J., Jayatilaka, D. & Spackman, M. A. (2021). *J. Appl. Cryst.* **54**, 1006–1011. 10.1107/S1600576721002910 PMC8202033 34188619

[bb30] Tan, S. L., Jotani, M. M. & Tiekink, E. R. T. (2019). *Acta Cryst.* E**75**, 308–318. 10.1107/S2056989019001129 PMC6399703 30867939

[bb31] Zhou, C.-H. & Wang, Y. (2012). *Curr. Med. Chem.* **19**, 239–280. 10.2174/092986712803414213 22320301

